# PERORAL ENDOSCOPIC MYOTOMY FOR ACHALASIA: SAFETY PROFILE, COMPLICATIONS AND RESULTS OF 94 PATIENTS

**DOI:** 10.1590/0102-672020230066e1784

**Published:** 2023-12-08

**Authors:** Francisco Paulo Ponte PRADO, Ivens Filizola Soares MACHADO, Maria Paula Lopes Ponte PRADO, Renato Bruno Cavalcante LEITE, Samuel Magalhães GURGEL, José Walter Feitosa GOMES, José Huygens Parente GARCIA

**Affiliations:** 1 Hospital Geral Dr. César Cals, Secretary of Health, Fortaleza (CE), Brazil;; 2 Instituto Dr. José Frota, Fortaleza (CE), Brazil;; 3 Hospital Universitário Walter Cantídio, Fortaleza (CE), Brazil.

**Keywords:** Deglutition Disorders, Endoscopy, Digestive System, Esophageal Achalasia, Myotomy, Transtornos de Deglutição, Endoscopia do Sistema Digestório, Acalasia Esofágica, Miotomia

## Abstract

**BACKGROUND:**

Achalasia is an esophageal motility disorder, with clinical presentation of dysphagia and regurgitation. This is a chronic condition with no cure. Current treatment options aim to reduce lower esophageal sphincter tone by pharmacological, endoscopic or surgical means, with the aim of improving patients’ symptoms. Peroral endoscopic myotomy (POEM) is an alternative endoscopic surgery to Heller cardiomyotomy, in which the procedure is performed orally, by endoscopy, offering efficacy comparable to surgical myotomy, with relative ease and minimal invasion, without external incisions.

**AIMS:**

To study the safety of POEM by analyzing its results, adverse events and perioperative complications and the main ways to overcome them, in addition to evaluating the effectiveness of the procedure and the short-term postoperative quality of life.

**METHODS:**

A qualitative and quantitative, observational and cross-sectional study that analyzed patients who underwent the POEM in a reference center, from December 2016 to December 2022, maintaining the technical standard of pre-, peri- and postoperative protocol.

**RESULTS:**

A total of 94 patients were included in the study, and only three had postoperative complications. The average early postoperative Eckardt score was 0.93 and the late 1.40, with a mean improvement of 7.1 in early results and 6.63 in late results (p<0.05).

**CONCLUSIONS:**

POEM can be reproduced with an excellent safety profile, significant relief of symptoms and improvement in esophageal emptying, and in quality of life.

## RESUMO

**RACIONAL:**

A acalasia é uma desordem da motilidade esofágica, com apresentação clínica de disfagia e regurgitação, uma condição crônica sem cura. As opções de tratamentos atuais visam reduzir o tônus do esfíncter esofagiano inferior (EEI) por meios farmacológicos, endoscópicos ou cirúrgicos, com o objetivo de aliviar os sintomas dos pacientes. A miotomia endoscópica peroral, sigla do inglês (POEM) é uma cirurgia endoscópica alternativa à cardiomiotomia a Heller (CH), em que a miotomia é realizada por via oral, por endoscopia, oferecendo eficácia comparável a miotomia cirúrgica, com relativa facilidade e mínima invasão, sem incisões externas.

**OBJETIVOS:**

Analisar a segurança do POEM analisando seus resultados, eventos adversos, complicações perioperatórias e suas principais formas de contorná-los, além de avaliar a eficácia do procedimento e a qualidade de vida pós-operatória, em curto prazo.

**MÉTODOS:**

Estudo qualitativo e quantitativo, observacional e transversal avaliando pacientes que realizaram o POEM em um centro de referência, entre dezembro de 2016 até dezembro de 2022, mantendo padrão técnico de preparo pré, peri e pós-operatório.

**RESULTADOS:**

Foram incluídos no estudo 94 pacientes e apenas 3 apresentaram complicações pós-operátorias. O escore de Eckardt médio precoce pós-operatório foi de 0,93 e o tardio de 1,40, com melhora média de 7,1 em resultados precoces e de 6,63 em resultados tardios (p<0,05).

**CONCLUSÕES:**

O POEM pode ser reproduzido com excelente perfil de segurança, alívio significativo dos sintomas e melhora do esvaziamento do esôfago, oferecendo melhora na qualidade de vida.

## INTRODUCTION

A chalasia is a motility disorder of the esophagus characterized by a lack of peristalsis within the body of the esophagus and reduced or absent relaxation of the lower esophageal sphincter (LES). It typically has an idiopathic origin^3^. This condition is marked by the degeneration of the myenteric plexus, leading to a deficiency in the relaxation of the lower esophageal sphincter and disorganized peristalsis in the esophageal body^29^. Achalasia is a chronic ailment for which there is currently no cure. The primary symptomatic manifestation of this motility disorder is the classic presentation of difficulty swallowing both solids and liquids, often accompanied by regurgitation of undigested food or saliva^27^. While achalasia is considered rare, it is the most prevalent primary esophageal motility disorder, with an annual incidence of approximately 1.6 case per 100 thousand individuals and a prevalence rate of approximately 10.8 cases per 100 thousand individuals. It affects both sexes equally and typically presents around the age of 50^23^. In addition to the idiopathic form of achalasia, there are other types of secondary achalasia, such as Chagas’ disease, which is caused by the parasite *Trypanosoma cruzi* ^29^.

### Diagnosis

Barium swallow test plays a crucial role as a diagnostic test for assessing the esophagus’ morphology, including its diameter and alignment. When it comes to diagnosing achalasia, contrast examination reveals some characteristic findings, which include the absence of the gastric bubble, a tapering of the distal esophagus resulting in a “bird’s beak” configuration, and proximal organ dilation, occasionally accompanied by the presence of hydroaerial levels^17^.

The Rezende and Mascarenhas Classification (1982) utilizes an esophagogram to gauge the severity of achalasia, serving as one of the criteria for determining the appropriate treatment. This classification system categorizes achalasia into four grades: Grade I (unchanged caliber of the esophagus, measuring less than 4 cm, with minimal contrast retention); Grade II (moderate increase in esophageal caliber, ranging from 4 to 7 cm, accompanied by contrast retention and the presence of tertiary waves); Grade III (significant enlargement of the esophageal caliber, measuring between 7 and 10 cm, often associated with hypotonia); Grade IV (corresponds to dolico-megaesophagus, characterized by an esophageal caliber exceeding 10 cm)^21^.

The Eckardt symptom score is a scoring system that takes into account the frequency of each of the four main symptoms of achalasia, including dysphagia, regurgitation, retrosternal pain and weight loss. Each symptom is scored on a scale of 0 to 3, resulting in total possible scores between 0 and 12, with higher scores indicating more symptomatic disease. Scores of 3 or less are considered a good response to treatment^5^.

High-resolution manometry is essential in the management of achalasia with endoscopic myotomy, since using the Chicago classification the extent of myotomy can be standardized according to type I, aperistalsis; type II, pan-pressurization; and type III, distal pressurization^4^.

Endoscopic examination is an integral part of achalasia investigation and can provide a diagnostic confirmation in cases of stasis and resistance at the lower sphincter. Care should be taken in cases of pseudo-achalasia, as it mimics the condition. It is also useful to rule out other diagnoses, such as neoplasms and esophagitis^7^.

Current treatment options for this condition aim to reduce the tone of the LES by pharmacological, endoscopic or surgical means. The goals in treating achalasia are to relieve the symptoms of dysphagia, to improve esophageal emptying and to prevent the organ from becoming more dilated over the years^27^.

Different oral pharmacological agents have been used in the management of achalasia, including calcium channel blockers (nifedipine), long-acting nitrates (isosorbide dinitrate), phosphodiesterase-5 inhibitors (sildenafil), anticholinergics (atropine and dicyclomine), and beta-adrenergic agonists (terbutaline). Calcium channel blockers and long-acting nitrates are the most commonly used^27^. These agents act by facilitating esophageal emptying, reducing tone, and causing relaxation of the LES. Oral agents, however, are among the least effective treatment options due to their mechanisms of action, providing only transient benefits and carrying a significant potential for side effects^27^.

Another treatment option for achalasia involves injecting botulinum toxin directly into the LES using upper digestive endoscopy. Botulinum toxin causes presynaptic inhibition of acetylcholine release and blocks unopposed excitatory cholinergic stimulation of the LES, leading to paralysis of the sphincter due to neuronal inhibition, but with no effect on resting muscle tone, which is mainly driven by myogenic influence^27^. As a result, the overall effect of botulinum toxin injection is an approximate 50% reduction in LES tone. This reduction is usually sufficient to facilitate esophageal emptying and provides relief from symptoms in nearly two-thirds of patients^8^.

Pneumatic dilation involves the use of polyethylene balloons of varying sizes, which are gradually inflated to cause the rupture of the circular muscle fibers in the LES. Pneumatic dilation is currently the most widely used non-surgical option for treating achalasia^27^. For most patients, the initial dilation is conducted using a 3.0 cm balloon, followed by a symptomatic and objective assessment four to six weeks later. If patients continue to experience symptoms, further dilation with a balloon of a similar size can be considered. Studies have consistently demonstrated the effectiveness of pneumatic dilation, with some reporting that it provides good to excellent relief from symptoms in as many as 93% of patients^27,28^.

Pneumatic dilation has a risk of esophageal perforation with a median rate of 1.9% in the hands of experienced operators^6,28^. In addition, the risk of perforation may be lower in serial pneumatic dilatations, but they are less effective in young men under the age of 45 due to their thicker LES muscles.

Additional complications associated with pneumatic dilation include the development of gastroesophageal reflux disease (GERD) in approximately 15–35% of patients^19^. Prolonged post-dilation chest pain, intramural hematoma and traumatic diverticula have also been described after pneumatic dilations^6^.

A European randomized controlled trial comparing the efficacy of pneumatic dilation versus laparoscopic HM showed no significant difference in the efficacy of the two modalities after two years of follow-up^2^. The success rate at two years, according to the Eckardt score, was 86% after pneumatic dilation and 90% after laparoscopic HM, with no statistically significant difference (p=0.46). In addition, there were no significant differences in post-procedure LES pressure, barium contrast column height or quality of life between the two groups. A long-term follow-up study showed similar efficacy, even at 5 years, although 25% of patients undergoing pneumatic dilatation required further dilatation^16^.

POEM is an endoscopic surgical alternative to open or laparoscopic HM^1^. In this procedure, myotomy is performed orally using an endoscope to create a submucosal tunnel. POEM offers efficacy comparable to surgical myotomy but with minimal invasiveness and relative ease, without requiring external incisions^18^.

The concept of endoscopic myotomy for achalasia was initially reported in 1980, but there were no subsequent reports on the seven cases performed at that time. In 2009, Inoue et al. in Japan refined the technique and coined the term “POEM”^9^. This innovative approach involves creating a submucosal tunnel followed by myotomy of the esophageal circular layer, which differs from MH, where myotomy targets both the longitudinal and circular layers. POEM is considered a safe procedure, comparable to the safety profile of laparoscopic Heller cardiomyotomy^24^.

This study evaluates the safety of POEM by analyzing its outcomes, adverse events, perioperative complications, and strategies for preventing them. Additionally, the study assesses the effectiveness of the procedure and its impact on postoperative quality of life.

## METHODS

This is a qualitative and quantitative, observational and cross-sectional study, with a review of medical records and carried out in a tertiary hospital belonging to the *Sistema Único de Saúde* (SUS), Brazil’s health system, in the state of Ceará, located in northeastern Brazil.

The study included all patients who received a confirmed diagnosis of esophageal achalasia based on the clinical presentation, digestive endoscopy, esophagogram and esophageal manometry and who underwent the POEM procedure, from December 2016 to December 2022, operated on by the same team, maintaining the same pre-, peri- and post-operative protocol, following these steps:

Step 00 – Pasty diet for 48 hours, clear liquids without residue for 24 hours and fasting for 8 hours before the procedure.

Step 1 – Test of the digestive endoscopy equipment, using Olympus Exera II CV-180 with CO2 system, 150 or 180 series endoscope.

Step 2 – General anesthesia with orotracheal intubation and antibiotic prophylaxis with ciprofloxacin 400 mg and metronidazole 500 mg single dose.

Step 3 – Esophageal assessment and lavage with distilled water using a distal attachment cap at the tip of the endoscope (endoscopes with larger working channels can be used in case of difficulty).

Step 4 – Localization of the cardia by measuring the distance to the upper dental arch.

**Figure 1:**
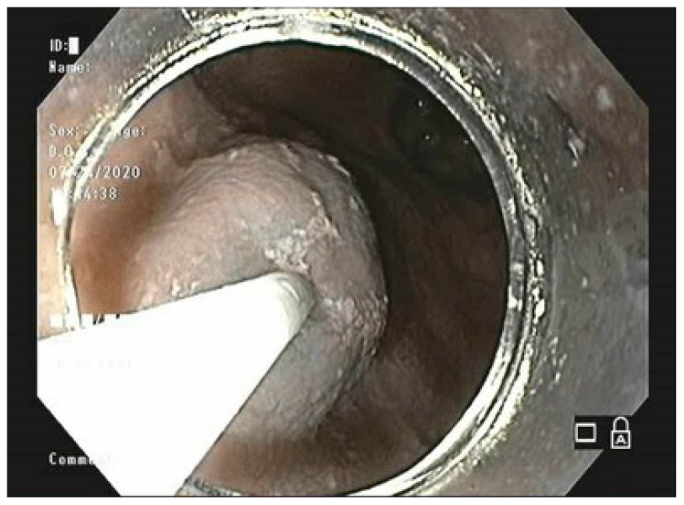
Making a bubble in submucosa.

Step 5 – Submucosal injection of saline solution with methylene blue into the right anterolateral wall, about 10 cm from the cardia, making a bubble in the submucosa (Figure 1).

**Figure 2:**
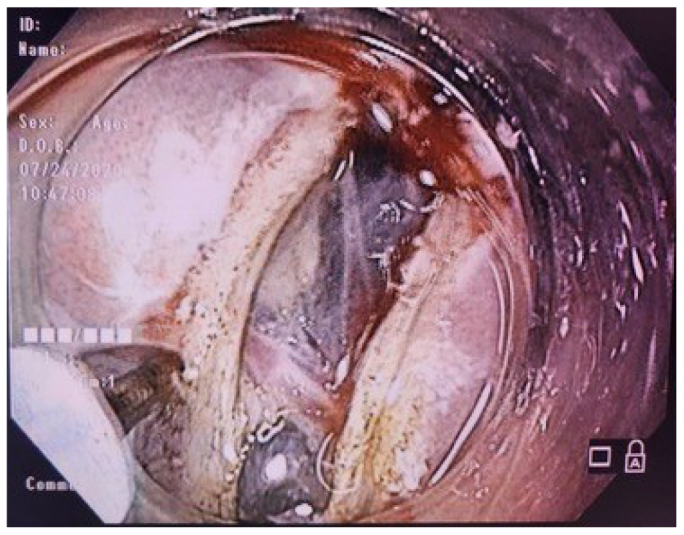
Excision of the mucosa.

Step 06 – Excision of the mucosa measuring around 15 mm using a flush knife (Figure 2).

**Figure 3:**
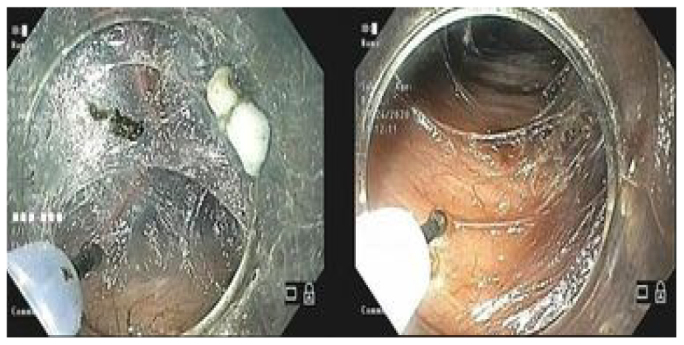
Making the submucosa tunnel.

Step 7 – Making the submucosal tunnel, detaching the submucosa from the circular muscle layer using a flush knife and injections of methylene blue solution (Figure 3).

**Figure 4:**
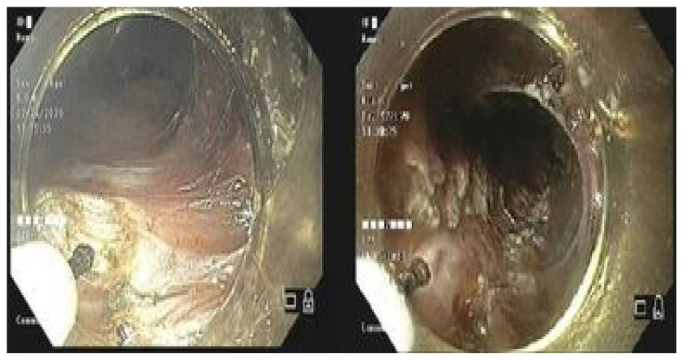
Myotomy.

Step 8 – Endoscopic myotomy starting 2–3 cm below the excision of the mucosa up to 3 cm below the cardia (Figure 4).

**Figure 5:**
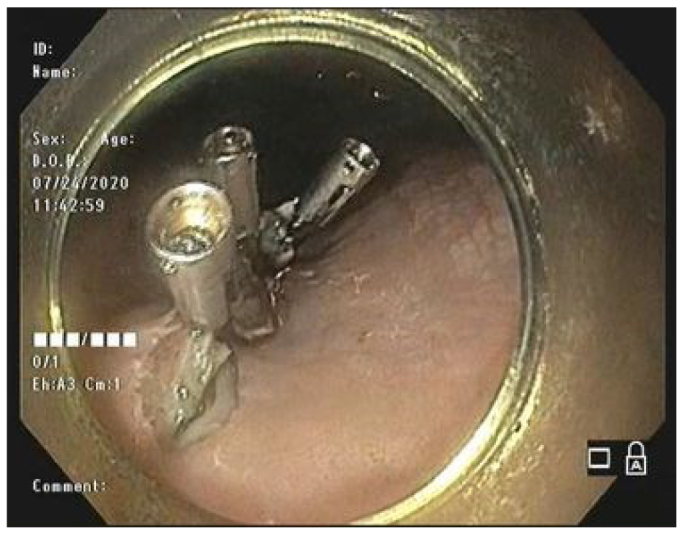
Closure of the mucosal excision.

Step 9 – Review of hemostasis and closure of the mucosal excision (Figure 5).

Step 10 – Endoscopic assessment of possible intraoperative events, with subsequent interventions according to medical criteria.

Step 11 – Fasting was recommended for 24 hours, followed by a restricted liquid diet on the first postoperative day, full liquid on the second, pasty on the third, fourth and fifth. Pantoprazole 40 mg twice a day was prescribed for 60 days.

Step 12 – Follow-up was carried out with a medical consultation and digestive endoscopy assessing symptoms, the Eckardt score and endoscopic findings at three, six, 12 months and annually after the procedure.

Gender, age, prior achalasia treatments, including the type of treatment, Rezende’s radiological classification from preoperative barium esophagograms, preoperative Eckardt scores, the presence of adverse events, and intra- and peri-operative complications were assessed.

Postoperative complications were classified according to the Clavien-Dindo criteria. Length of hospital stay, perioperative mortality and the need for readmissions were also assessed.

Late follow-up was carried out through medical consultations assessing symptoms and the result of the most recent upper digestive endoscopy. The Eckardt scores were evaluated early after the procedure, at three to six months post-surgery, and, subsequently, after one year or more.

Patients with incomplete medical records, under 15 years of age, who refused to take part in the study and patients in whom endoscopic surgery was not performed for technical reasons were excluded. Patients with a history of endoscopic dilation, botulinum toxin injection or a history of laparoscopic esophageal myotomy before POEM were not considered exclusion criteria.

### Statistical analysis

Data was collected retrospectively to compare pre- and post-procedure parameters and statistical analysis was performed. Data is presented as mean ± standard deviation. Student’s paired t-test was used for continuous variables and the proportion test for categorical variables. A p-value <0.05 was considered statistically significant. The study was approved by the Ethics Committee of Hospital Geral Doutor César Cals (nº 67147423.1.0000.5041).

## RESULTS

The study included 94 patients, with 55 females and 39 males, ranging in age from 17 to 90 years. The mean age was 51.05 years, with a median of 53 years. Rezende’s classification was applied to only 67 patients, of which 13 (19.3%) were grade I, 31 (46.3%) grade II, 18 (26.9%) grade III and five (7.5%) grade IV.

Adverse events were documented in 47 patients (50%) during the procedure. These events included subcutaneous emphysema in 29 patients (30.9%), pneumoperitoneum in nine (9.6%), bleeding in four (4.3%), pneumothorax in three (3.2%), and inadvertent injury to the tunnel mucosa in five (5.3%). Subcutaneous emphysema was self-limiting due to the use of CO_2_, which is rapidly absorbed by the tissues. Pneumothorax and pneumoperitoneum were treated by inserting a 14G needle into the fifth intercostal space of the affected hemithorax and into the left hypochondrium, respectively, keeping it open until the end of the procedure. Patients with tunnel mucosa rupture underwent treatment through endoscopic closure with a clip. Among those who experienced bleeding, three were managed through cauterization using the flush knife and/or a hemostatic forceps, and one patient needed treatment with a Sengstaken-Blakemore balloon.

Two patients required extended hospitalization, one for a total of nine days and the other for 14 days due to pneumothorax. Additionally, another patient needed readmission due to the development of pleural empyema, which was successfully treated through lung decortication.

Out of the 94 patients included in the study, only three experienced post-operative complications: one patient was classified as Clavien-Dindo II (respiratory infection), one as Clavien-Dindo IIIa (pneumothorax), and one as Clavien-Dindo IIIb (pleural empyema).

The preoperative Eckardt score ranged from 4 to 12, with an average of 8.03.

The early postoperative Eckardt score assessed in 42 patients had a mean of 0.93. The late postoperative Eckardt score assessed in 39 patients had a mean of 1.40, after 47 months of late follow-up. Therefore, there was an average improvement of 7.1 in the early results and 6.63 in the late results, with statistical significance (p<0.05) (Table 1).

**Table 1:** Results of the Eckardt score in the preoperative period, and in the early and late postoperative periods.

	Mean	n	Standard deviation (SD)	Standard error of mean	p-value
Pre-op Eckardt	7.86	42	2.495	0.385	<0.001
Early Eckardt	0.90	42	1.122	0.173	
Pre-op Eckardt	7.64	39	2.444	0.391	<0.001
Late Eckardt	1.44	39	1.353	0.217	

PRE-OP: pre-operative; SD: standard deviation.

It was possible to evaluate postoperative upper digestive endoscopy results in 46 patients, on average, 22.3 months after the surgery. Among them, 28 (60.9%) showed no esophagitis, seven (15.2%) had Los Angeles grade A esophagitis, seven (15.2%) had Los Angeles grade B esophagitis, three (6.5%) had Los Angeles grade C esophagitis, and one (2.1%) had Los Angeles grade D esophagitis. Additionally, 31 (67.4%) had no esophageal residue, seven (15.2%) had salivary esophageal residue, five (10.8%) had liquid food esophageal residue, and three (6.5%) had solid-pasty food esophageal residue.

## DISCUSSION

Currently, there is no standard technique adopted by all health centers for the treatment of achalasia, with variations in periprocedural management between them. Despite the good results, excellent efficacy and safety of POEM are commonly reported, clinical success rates and adverse events vary between healthcare providers^15,20^. In this study, the results of the procedure were evaluated with a description of the technique used for reproduction and comparison with other healthcare centers.

Clinical remission after POEM ranges from 82 to 100%, with a reduction in the Eckardt score and a decrease in LES pressure^19,24^. An Eckardt score of 3 or less has been achieved in around 98% of cases, according to a meta-analysis^1^. A cohort of more than 500 patients, with at least three years of follow-up, showed a success rate in maintaining symptom relief of 88.5%^24^.

The guideline from the American College of Gastroenterology recommends and supports the use of POEM as a safe treatment option for achalasia patients, even those who have previously undergone Heller cardiomyotomy or pneumatic dilation. Some meta-analyses, involving over one thousand patients, have shown the short-term efficacy of POEM (within one year), including reductions in the Eckardt score and LES pressure^19,24^.

In a review of laparoscopic HM, 0.6% of patients reported wound complications, 1.8% general complications and 2.4% major complications. The readmission rate was 3.1% and the reoperation rate was 2.3%. The 30-day mortality rate was 0.3%. The average operative time was just over 2 hours (141.6±63.4 min)^22^. A meta-analysis comparing patients who underwent POEM versus laparoscopic HM revealed that neither group experienced serious adverse events. Minor adverse events were more common in the laparoscopic group, whereas major events were observed more frequently in the POEM group^13^.

The main short-term adverse events of POEM include subcutaneous emphysema, pneumoperitoneum and pneumomediastinum^19^. Conservative treatment allows for rapid recovery, and such events have little influence on long-term prognosis. Another common adverse event is perforation, which in the case of the mucosa can be closed during the procedure using endoscopic clips^10^.

The present study demonstrates the excellent safety profile of POEM with significant symptom relief, improved esophageal emptying and a significant reduction in symptoms, offering an improvement in the patient’s quality of life. Common adverse events such as pneumoperitoneum and subcutaneous emphysema are easily managed if clinically significant, making POEM a safe procedure. Other factors such as operator experience and myotomy route may be additional factors responsible for the lower rate of these adverse events, which could be analyzed in further studies. Pneumothorax was the most serious complication and should be avoided. Among the various data analyzed in this study, one is very relevant: the mortality rate (postoperative complication Clavien-Dindo V) after POEM was zero, which is in line with other studies, as shown in the table below (Table 2).

**Table 2:** Comparison of the Clavien-Dindo score in literature studies.

Publications	n	CDI (%)	CDII (%)	CDIIIa (%)	CDIIIb (%)	CDIV and CDV
Meng et al.^14^	32	0	0	0	0	0
Ling et al.^11^	87	1 (1.1)	0	2 (2.3)	0	0
Liu et al.^12^	35	1 (2.8)	0	0	0	0
Zhang & Linghu^30^	32	4 (12.5)	0	2 (6.2)	0	0
Teitelbaum et al.^26^	41	7 (17)	0	0	1 (2.4)	0
Tang et al.^25^	67	2 (3)	0	0	0	0
Total	294	15 (5.1)	0	4 (1.3)	1 (0.3)	0
This study	94	0	1 (1)	1 (1)	1 (1)	0

CD: Clavien-Dindo.

This study has some limitations, such as the absence of some data and better stratification of the patient profile. Additional prospective and multicenter studies are needed to establish and standardize the technique.

## CONCLUSIONS

The POEM procedure can be reproduced safely, resulting in significant symptom relief, enhanced esophageal emptying and improved patient quality of life. However, additional prospective and multicenter studies are needed to establish and standardize the technique.
